# Type IV pilus retraction enables sustained bacteremia and plays a key role in the outcome of meningococcal sepsis in a humanized mouse model

**DOI:** 10.1371/journal.ppat.1009299

**Published:** 2021-02-16

**Authors:** Jean-Philippe Barnier, Daniel Euphrasie, Olivier Join-Lambert, Mathilde Audry, Sophia Schonherr-Hellec, Taliah Schmitt, Sandrine Bourdoulous, Mathieu Coureuil, Xavier Nassif, Mohamed El Behi

**Affiliations:** 1 Université de Paris, Faculté de Médecine, Paris, France; 2 Institut Necker Enfants-Malades, Inserm U1151, CNRS UMR 8253, Paris, France; 3 Service de microbiologie, Assistance Publique–Hôpitaux de Paris. Centre–Université de Paris, Hôpital Necker Enfants Malades, Paris, France; 4 Service de chirurgie reconstructrice et plastique, Groupe Hospitalier Paris Saint-Joseph, Paris, France; 5 Institut Cochin, Inserm U1016, CNRS UMR 8104, Paris, France; University of Oxford, UNITED KINGDOM

## Abstract

*Neisseria meningitidis* (the meningococcus) remains a major cause of bacterial meningitis and fatal sepsis. This commensal bacterium of the human nasopharynx can cause invasive diseases when it leaves its niche and reaches the bloodstream. Blood-borne meningococci have the ability to adhere to human endothelial cells and rapidly colonize microvessels. This crucial step enables dissemination into tissues and promotes deregulated inflammation and coagulation, leading to extensive necrotic purpura in the most severe cases. Adhesion to blood vessels relies on type IV pili (TFP). These long filamentous structures are highly dynamic as they can rapidly elongate and retract by the antagonistic action of two ATPases, PilF and PilT. However, the consequences of TFP dynamics on the pathophysiology and the outcome of meningococcal sepsis *in vivo* have been poorly studied. Here, we show that human graft microvessels are replicative niches for meningococci, that seed the bloodstream and promote sustained bacteremia and lethality in a humanized mouse model. Intriguingly, although pilus-retraction deficient *N*. *meningitidis* strain (Δ*pilT*) efficiently colonizes human graft tissue, this mutant did not promote sustained bacteremia nor induce mouse lethality. This effect was not due to a decreased inflammatory response, nor defects in bacterial clearance by the innate immune system. Rather, TFP-retraction was necessary to promote the release of TFP-dependent contacts between bacteria and, in turn, the detachment from colonized microvessels. The resulting sustained bacteremia was directly correlated with lethality. Altogether, these results demonstrate that pilus retraction plays a key role in the occurrence and outcome of meningococcal sepsis by supporting sustained bacteremia. These findings open new perspectives on the role of circulating bacteria in the pathological alterations leading to lethal sepsis.

## Author summary

Invasive meningococcal diseases remain a major cause of fatal sepsis. A specific feature of *Neisseria meningitidis* is its ability to colonize the blood microvessels in a type IV pilus (TFP)-dependent mechanism. TFP are filamentous appendages that undergo retraction through a mechanism dependent on the PilT ATPase. Here, we assess the role of TFP retraction in the pathophysiology of meningococcal infection using a humanized model of SCID mice grafted with human skin. We show that human skin graft microvessels are replicative niches for *N*. *meningitidis* that promote sustained bacteremia and subsequent lethality. Intriguingly, although pilus retraction-deficient *N*. *meningitidis* (Δ*pilT*) efficiently colonizes human grafts, this mutant strain did not promote sustained bacteremia nor induce mouse lethality. This drastic decrease in virulence was not due to a decreased inflammatory response or to a defect in bacterial clearance by the innate immune system. Rather, we demonstrate that pilus retraction was crucial to obtain a sustained bacteremia by allowing the release of bacteria from colonized microvessels and that lethality was directly linked to sustained bacteremia. Altogether, these data demonstrate the important role of pilus retraction in meningococcal pathogenesis and infection outcome and open new questions regarding the impact of circulating bacteria on the host during lethal sepsis.

## Introduction

*Neisseria meningitidis* (the meningococcus) is a Gram-negative extracellular bacterium whose ecological niche is the human nasopharynx [1]. In most cases, *N*. *meningitidis* is asymptomatically carried and lives in the nasopharyngeal mucus [2,3]. However, *N*. *meningitidis* is also responsible for invasive meningococcal diseases (IMD). The pathology is initiated when the bacterium crosses the nasopharyngeal epithelium, reaches the bloodstream and causes bacteremia leading to sepsis and meningitis. A central step in the pathophysiology of meningococcal infections is the extensive adhesion of meningococci to the endothelium in a process referred to as vascular colonization [4–7]. This colonization induces thrombotic events, deregulated inflammation and a leakage syndrome [6,8,9] leading to the development of *purpura fulminans* in the most severe forms [10]. At the early stage of IMD, clinical evidences shows that 42% to 70% of patients have cutaneous purpuric lesions that are known to contain viable meningococci [11–13]. At later time-points of infection, the massive disseminated microvascular colonization throughout the body in patients suffering from *purpura fulminans* is associated with a high bacterial load in the blood [14–16]. Moreover, vascular colonization is still present in the rare forms of chronic meningococcemia [17,18].

The capacity of pathogenic encapsulated meningococci to interact tightly with human endothelial cells relies on the expression of type IV pili (TFP) [19–21]. These long filamentous appendages are associated with many features such as adhesion to host cells, DNA uptake, twitching motility and bacteria-bacteria interactions [6,20,22–26]. Using infected SCID mice grafted with human skin, which recapitulates the tissue lesions observed in patients with IMD, earlier works demonstrated that TFP are also required for meningococcal interaction with microvessels *in vivo* and subsequent vascular colonization, leading to tissue damage and lethality [6,20,27,28]. Neisserial TFP are composed of a core pilin subunit (PilE) and minor pilins (PilV, PilX, ComP) assembled into an helical structure [29,30]. Meningococcal TFP mediate bacterial aggregation, a process stabilized by the minor pilin PilX [24,31]. The biosynthesis of functional TFP involves a complex machinery comprising many proteins encompassing pilins and several accessory proteins [32]. Among them, the outer-membrane associated PilC proteins are encoded in *N*. *meningitidis* by *pilC1* and *pilC2* genes. PilC1 and PilC2 have similar but non-redundant functions in piliation. PilC1 is required for TFP adhesiveness, as a *pilC1* mutant is piliated but non-adhesive [33–35]. PilC1 is proposed to be the tip-located TFP adhesin of *N*. *meningitidis* [36]. TFP are highly dynamic structures as they can elongate and retract through the antagonist action of two ATPases (PilF and PilT, respectively) that are associated with the inner membrane. PilC proteins are involved in TFP dynamics by antagonizing PilT-mediated pilus retraction [34]. Moreover, PilT can downregulate the expression of PilC1 and negatively control the adhesiveness of the TFP by a mechanism independent of piliation [37]. Absence of pilus retraction, by deletion of the *pilT* gene, causes a hyper-piliated and hyper-aggregative phenotype with increased adhesion to host cells whereas twitching motility and natural competence are lost [26,38]. In addition, cooperative retraction of bundled TFP enables nanonewton force generation that affects bacteria-bacteria and bacteria-host interactions [39]. *In vivo*, TFP retraction appears to be required for the adaptation of *N*. *meningitidis* to the constrained geometry of the microvessels [40]. However, whether TFP retraction plays a role in the pathophysiology and the outcome of meningococcal sepsis remains unknown.

Here, we took advantage of the validated model of SCID mice grafted with human skin to assess the role of TFP retraction in the pathogenesis of *N*. *meningitidis*. We demonstrate that although a PilT-deficient meningococcal strain (Δ*pilT*, pilus retraction deficient strain) adhered and colonized human graft similarly to the wild-type (WT) strain, the mutant failed to promote a sustained bacteremia and mouse lethality. This effect was not due to reduced inflammatory response nor altered clearance of this mutant by the immune system. TFP retraction was necessary to trigger the release of TFP-dependent bacteria-bacteria contacts that enables bacteria to detach from colonized microvessels, a process required for blood seeding and sustained bacteremia. Furthermore, we show that lethality directly results from the ability of the WT strain to promote a sustained bacteremia. Altogether, these results demonstrate that pilus retraction plays a key role in the pathogenesis of *N*. *meningitidis* by promoting sustained bacteremia leading to lethal sepsis.

## Results

### Human graft is a replicative niche for *Neisseria meningitidis*

**Fig 1:**
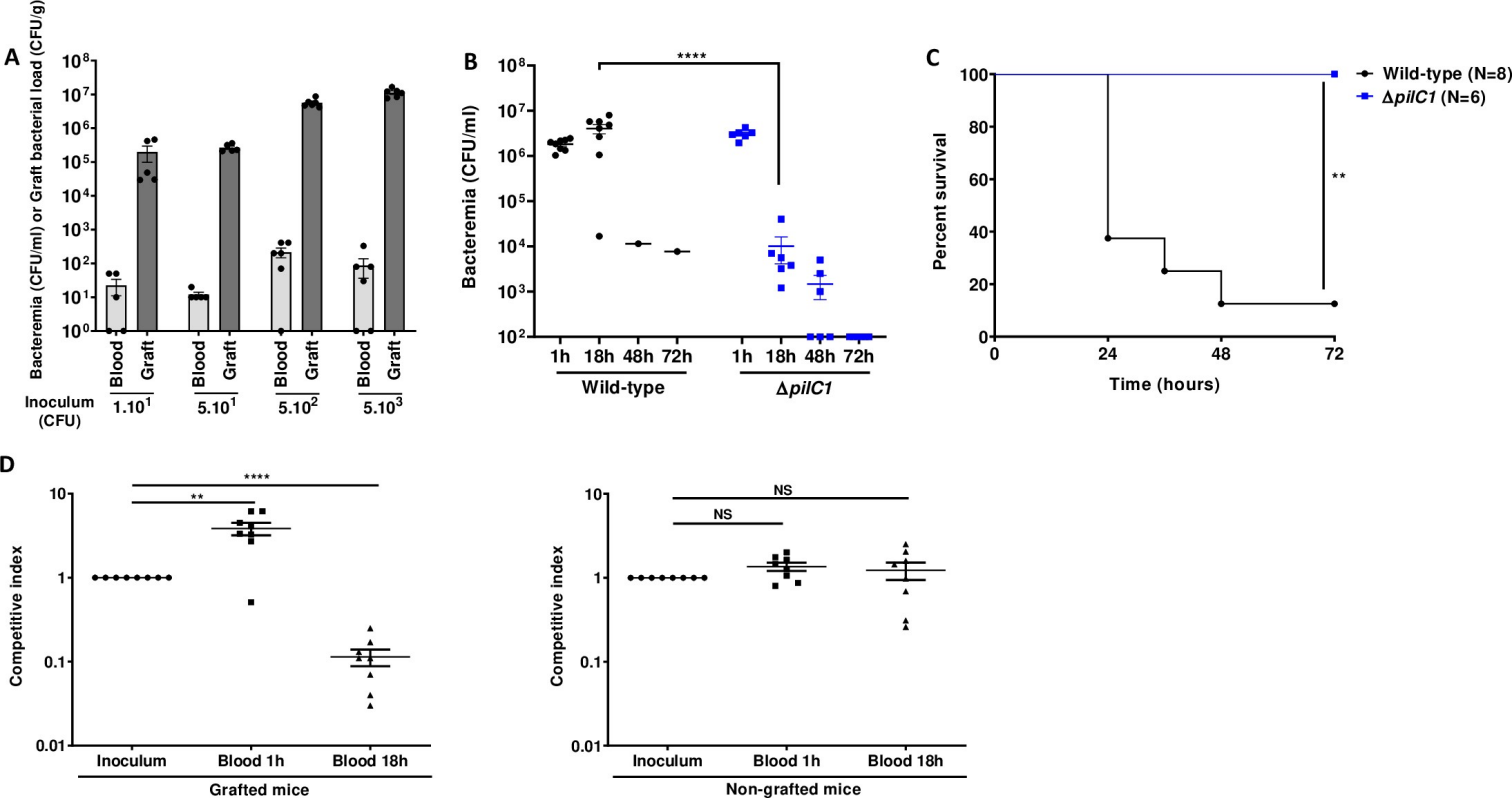
Human graft is a replicative niche for *Neisseria meningitidis* allowing sustained bacteremia. **(A)** Grafted mice were infected with a rising range of inocula: 1x10^1^, 5x10^1^, 5x10^2^, and 5x10^3^ CFU of WT *N*. *meningitidis*. Bacteremia and graft bacterial load were assessed at the time of sacrifice 18 h PI by quantitative culture. Bacterial counts are expressed in CFU/ml for blood and in CFU/g for graft. Two independent experiments, *n* = 5 or 6 mice per group, grafted with skin obtained from two different donors. Bars represent mean ± SEM. **(B)** Grafted mice were infected intravenously with 5x10^6^ CFU of WT *N*. *meningitidis* and its isogenic piliated non-adhesive mutant Δ*pilC1*. Bacteremia was measured at 1, 18, 48 and 72 h by culturing serial dilutions of blood samples on agar plates. Two independent experiments, *n* = 6 or 8 mice per group. Bars represent mean ± SEM, **** *p* < 0.0001, one-way ANOVA followed by multiple comparison test. **(C)** Kaplan-Meier plot showing the survival of infected grafted mice shown in panel B. Mice survival was assessed each day during 72 h. Two independent experiments, ** *p <* 0.01, two-sided log-rank Mantel-Cox analysis. **(D)** A competition assay between WT *N*. *meningitidis* and its isogenic piliated non-adhesive mutant Δ*pilC1* was performed on both grafted (left panel) and non-grafted (right panel) mice. Mice were infected IV with an inoculum of 1x10^7^ CFU total (a mixture of 5x10^6^ CFU of WT and 5x10^6^ CFU of Δ*pilC1* mutant). Competitive index, defined as the mutant/WT ratio within the output sample, divided by the corresponding ratio in the inoculum, was measured in the blood of mice at 1 h and 18 h PI by tail vein blood puncture. Two independent experiments, *n* = 8 mice per group. Bars represent mean ± SEM, NS *p* > 0.05; ** *p* < 0.01; **** *p* < 0.0001, one-way ANOVA followed by multiple comparison test.

Previous work in a model of SCID mice grafted with human skin suggested that human microvessels provide nutritional niches for colonizing bacteria [5]. To further investigate the role of human skin graft as a replicative niche for blood-borne meningococci during systemic infection, we infected human-skin grafted mice intravenously (IV) with increasing inocula of *N*. *meningitidis*, from 10 up to 5x10^3^ colony forming units (CFU), and determined the ability of the bacteria to colonize the graft and proliferate. As few as 10 bacteria were sufficient to heavily colonize the graft (**Fig 1A**). For all inocula tested, the number of CFU recovered in human grafts 18 h post-infection (PI) were 3 to 4 orders of magnitude higher than the number of bacteria injected IV (**Fig 1A**). These results clearly show that in this model, meningococcal colonization of human skin graft is highly efficient and support massive bacterial multiplication.

### TFP-dependent vascular colonization is required to maintain a sustained bacteremia

We next aimed at determining the origin of the bacteria present in the bloodstream of infected grafted mice. We monitored the level of bacteremia in animals infected IV with 5x10^6^ CFU of WT *N*. *meningitidis*, a lethal inoculum in grafted mice [5]. As expected, 7 out of 8 mice had a bacteremia of 10^6^ CFU/ml or above 18 h PI and died between 24 and 72 h. Only one mouse, which had a low bacteremia, survived (**Fig 1B and 1C**). All animals infected with a non-adhesive isogenic Δ*pilC1* strain survived and beyond 18 h exhibited a rapidly decreasing level of circulating bacteria (**Figs 1B and 1C and S1**). These results suggested that bacteria present in the bloodstream at late time-points of infection are mostly coming from bacteria multiplying within the human graft. To test this hypothesis, we performed a competition assay using the WT adhesive strain and the non-adhesive Δ*pilC1* mutant. We reasoned that there would be an enrichment in adhesive WT bacteria within the bloodstream at 18 h PI due to the ability of WT to adhere within the human graft and seed the bloodstream. We enumerated the bacterial load of the WT and the non-adhesive Δ*pilC1* mutant-strain in the blood and defined the competitive index as the mutant/WT ratio within the output sample, divided by the corresponding ratio in the inoculum. One-hour PI, in grafted mice infected with a mixture containing 5x10^6^ CFU of WT and 5x10^6^ CFU of Δ*pilC1* mutant, non-adhesive Δ*pilC1* bacteria showed an increased competitive index and were recovered at a greater number than WT bacteria (**Fig 1D, left panel**). On the other hand, at 18 h PI, the competitive index decreased by an order of magnitude, indicating that the Δ*pilC1* mutant was poorly recovered from the blood compared to WT (**Fig 1D, left panel**). As a control, we performed a similar competition assay in non-grafted animals where neither WT nor Δ*pilC1* mutants are able to adhere to mouse blood vessels. Under these conditions, both strains were similarly cleared from the blood and no difference in the competitive index was observed at 1 h or 18 h PI (**Fig 1D, right panel**). The apparent competitive advantage of the Δ*pilC1* mutant at 1 h PI is likely a consequence of the high tropism of WT bacteria for the human graft and the removal of WT bacteria from the bloodstream through rapid adhesion to the human graft microvessels, while Δ*pilC1* mutants remain in the bloodstream. Conversely, over the course of the infection, at 18 h PI, the adhesive WT meningococci multiply actively within the graft and are released into the bloodstream, thereby outcompeting the non-adhesive Δ*pilC1* meningococci. Together, these results demonstrate that TFP-dependent colonization of the human vessels was a prerequisite to sustained bacteremia and that WT bacteria, but not the non-adhesive Δ*pilC1* meningococci, likely seed the blood by bacteria detaching from microcolonies grown within the human graft.

### Pilus retraction is required to maintain a sustained bacteremia and to induce lethality

**Fig 2:**
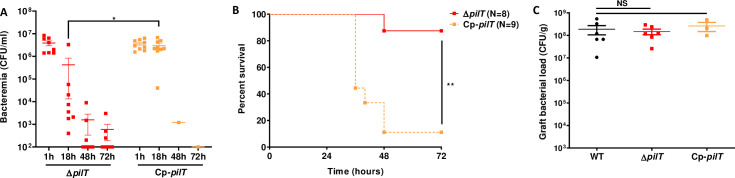
Pilus-retraction is required to establish sustained bacteremia and lethality. **(A)** Grafted mice were infected IV with 5x10^6^ CFU of *N*. *meningitidis*, Δ*pilT* mutant and its complemented Cp-*pilT* strain. Bacteremia of mice was measured at 1, 18, 48 and 72 h PI by culturing serial dilutions of blood samples on agar plates. Two independent experiments, *n* = 8 or 9 mice per group. Bars represent mean ± SEM, * *p* < 0.05, one-way ANOVA followed by multiple comparison test. **(B)** Kaplan-Meier plot showing the survival of infected grafted mice shown in panel A. Mice survival was assessed each day during 72 h. Two independent experiments, ** *p* < 0.01, two-sided log-rank Mantel-Cox analysis. **(C)** Grafted mice were infected IV with 5x10^6^ CFU of WT *N*. *meningitidis*, isogenic Δ*pilT* mutant, or complemented strain Cp-*pilT*. Graft bacterial load at 18 hours PI was measured by quantitative culture on agar plates. Two independent experiments for WT and Δ*pilT* strains and one experiment for complemented Cp-*pilT* strain, *n* = 3 or 6 mice per group. Bars represent mean ± SEM, NS *p >* 0.05, one-way ANOVA followed by multiple comparison test.

We next aimed to assess the role of pilus retraction in meningococcal pathogenesis using a pilus-retraction deficient mutant, Δ*pilT*, and a complemented Cp-*pilT* strain expressing the WT *pilT* allele (**Fig 2**). Similar to the WT strain, the Cp-*pilT* strain killed 8 out of 9 (89%) of the infected mice within 24 to 48 h. Conversely, the Δ*pilT* strain killed only 1 out of 8 (12,5%) of the infected animals when injected at a similar dose (**Fig 2B**). Accordingly, while bacteremia was sustained in animals infected with the Cp-*pilT* strain, as observed in animals infected with the WT strain, bacteremia decreased by several orders of magnitude in Δ*pilT* mutant-infected mice (**Fig 2A**). This effect was not due to a colonization defect as graft colonization at 18 h PI reached 1x10^8^ CFU/g in mice infected with Δ*pilT* strain, similarly to what was observed in grafts colonized by the WT and Cp-*pilT* strains (**Fig 2C**). Together, these results indicate that pilus retraction is required to maintain a sustained bacteremia in human skin-grafted animals and to induce lethality.

### *N*. *meningitidis* WT and Δ*pilT* trigger similar inflammatory responses and phagocytosis

**Fig 3:**
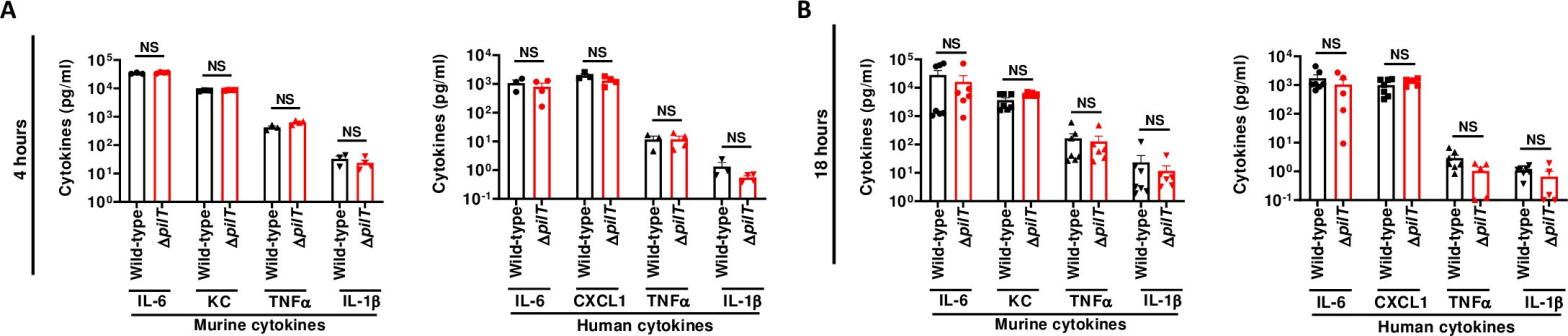
***N*. *meningitidis* WT and Δ*pilT* induce equivalent inflammatory responses (A, B)** Serum levels of proinflammatory mouse and human cytokines were measured in grafted mice infected IV with 5x10^6^ CFU of *N*. *meningitidis* WT strain or isogenic Δ*pilT* mutant at 4 h (A) and 18 h (B) post-infection using multiplex assays. Two independent experiments, *n* = 4, 5 or 6 mice per group. Bars represent mean ± SEM, NS *p >* 0.05, unpaired t-test.

We then addressed the question whether low bacteremia and higher survival observed with the Δ*pilT* mutant could result from a reduced inflammatory response and/or an increased clearance of this mutant by phagocytic innate immune cells. For this, we measured the level of murine and human pro-inflammatory cytokines in the blood of grafted mice infected with 5x10^6^ CFU of WT and Δ*pilT* at 4 and 18 h PI. Both WT and Δ*pilT* strains induced a canonical systemic inflammatory response with similar levels of murine IL-6, KC, TNFα and IL-1β cytokines, as well as a similar vascular inflammation in the infected skin grafts, as measured by human IL-6, CXCL1, TNFα and IL-1β cytokine levels (**Fig 3A and 3B**).

**Fig 4:**
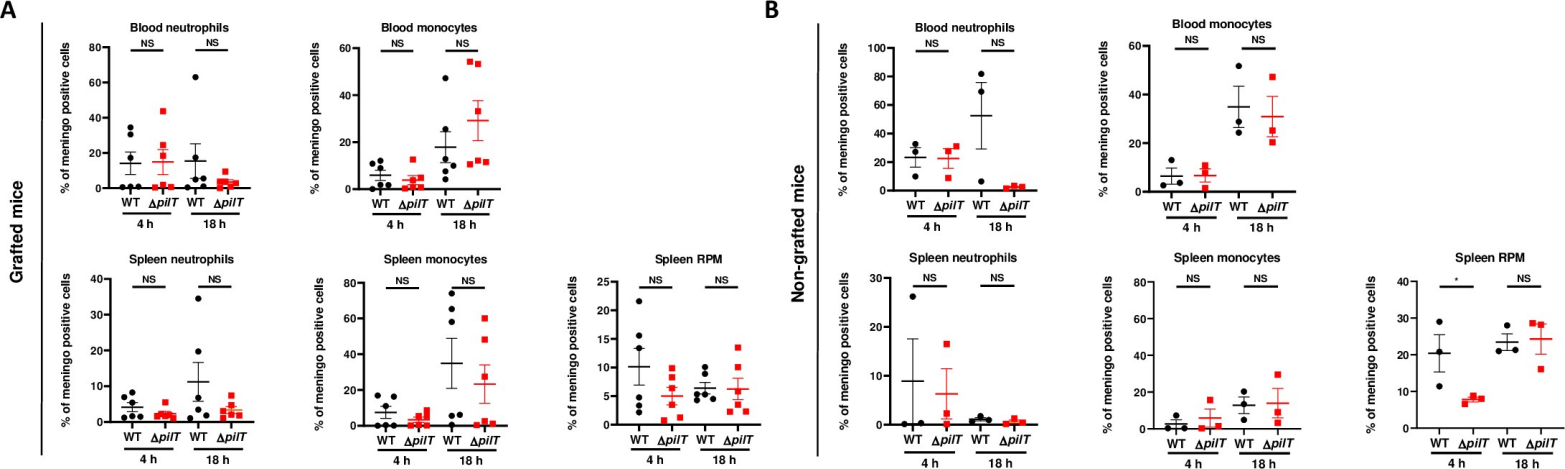
*N*. *meningitidis* WT and Δ*pilT* are similarly phagocytized. **(A, B**) *N*. *meningitidis* phagocytosis at 4 h and 18 h PI in the blood or spleen of grafted (A) and non-grafted (B) SCID mice. Neutrophils were identified as CD11b^+^Ly6G^hi^. Red pulp macrophages (RPM) were identified as CD11b^neg^ F4/80^hi^ and proinflammatory monocytes were identified as CD11b^+^ Ly6G^−^ Ly6C^hi^ F4/80^+^. Two independent experiments for grafted mice with *n* = 6 mice in each group (B) and one experiment for non-grafted mice with *n* = 3 mice in each group (A). Bars represent mean ± SEM, NS *p >* 0.05; * *p* < 0.05, one-way ANOVA followed by multiple comparison test.

Innate immune responses play a major role in the control of IMD as the timeframe of the infection is not compatible with a preponderant role of the adaptative immune response [10,13,41]. While SCID mice are devoid of adaptive immunity, they have a functional innate immune system. In particular, neutrophils, monocytes and red pulp macrophages (RPM) are present in the spleen of naïve SCID mice, although these mice lack marginal zone (MZ) macrophages and CD169^+^ metallophilic macrophages (**S2 Fig**) [42–44]. To assess the role of bacterial clearance in our model, we analyzed bacterial phagocytosis in different cell populations in the spleen and in the blood of infected animals by using a polyclonal antibody raised against *N*. *meningitidis*. We found that staining was barely detectable without permeabilization, indicating that the polyclonal antibody was specifically recognizing intracellular meningococci (**S3 Fig**). Upon infection with 5x10^6^ CFU of either WT or Δ*pilT* mutant in grafted SCID mice, phagocytosis of the Δ*pilT* mutant in the spleen by neutrophils, monocytes and RPM at 4 h and 18 h PI was not significantly different compared to that of the WT strain (**Fig 4A**). A similar result was observed in the blood where phagocytosis of WT and Δ*pilT* mutant by neutrophils and monocytes at 4 h and 18 h PI was equivalent (**Fig 4A**). In non-grafted SCID mice, phagocytosis of the two bacterial strains by blood and spleen phagocytes followed the same tendencies, with an exception for RPM phagocytosis of the Δ*pilT* mutant, which was slightly reduced as compared to that of WT at 4 h PI. However, this defect was not present at 18 h (**Fig 4B**). Hence, no significant difference was observed in the capacity of the innate immune cells of grafted mice to phagocyte the WT and Δ*pilT* mutant strains.

**Fig 5:**
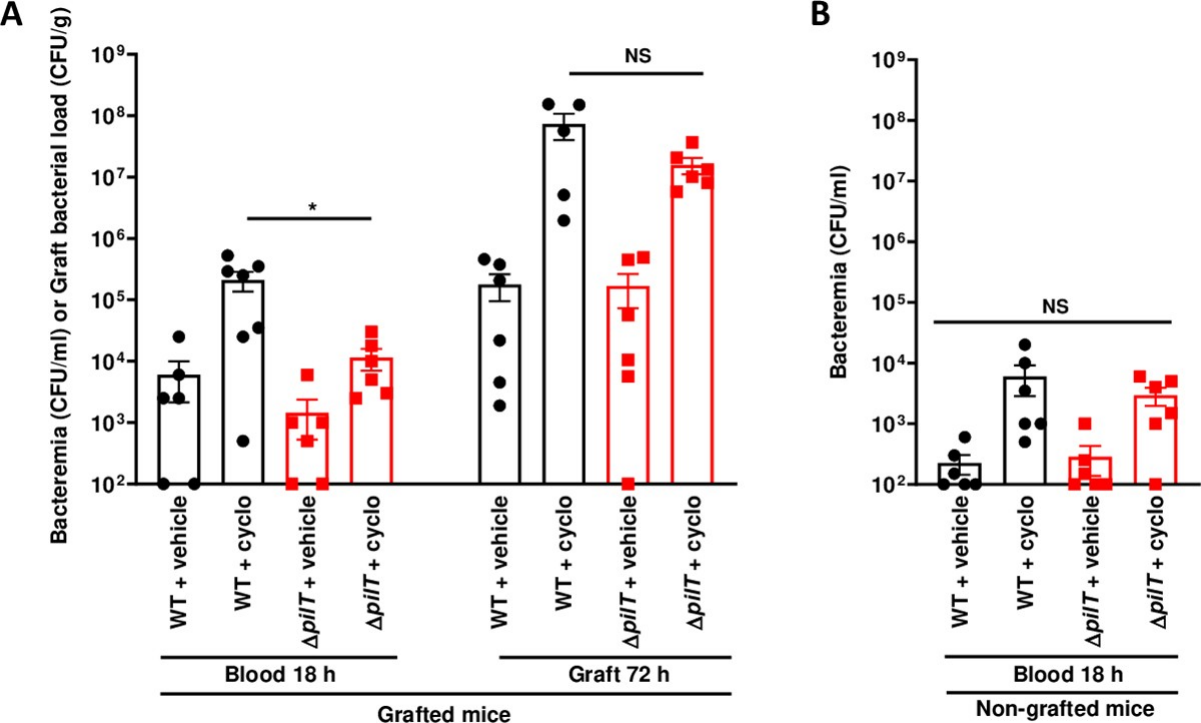
Monocytes and neutrophils actively control the bacteremia induced by WT or Δ*pilT* strains. **(A)** Grafted and **(B)** non-grafted SCID mice treated with cyclophosphamide (cyclo) and infected with 5x10^3^ CFU of WT *N*. *meningitidis* or Δ*pilT* mutant. Bacteremia was assessed at 18 h post-infection by blood puncture. Mice were sacrificed at 72 h post-infection and graft bacterial load was measured by quantitative culture. Two independent experiments, with *n =* 6 mice in each group. Bars represent mean ± SEM. NS *p >* 0.05; * *p* < 0.05, one-way ANOVA followed by multiple comparison test.

To further address the functional role of the innate immune system in bacterial clearance, SCID mice were treated with cyclophosphamide to deplete innate immune cells. Cyclophosphamide induced the depletion of neutrophils, monocytes, and splenic inflammatory monocytes but did not affect RPM (**S4 Fig**). Upon infection with a low inoculum of 5x10^3^ CFU of the WT strain, bacteremia at 18 h PI reached 6.0x10^3^ CFU/ml in non-treated mice whereas it reached 2.1x10^5^ CFU/ml, a 35-fold increase, in grafted animals treated with cyclophosphamide (**Fig 5A**). In contrast, bacteremia was significantly lower in animals infected with 5x10^3^ CFU of the Δ*pilT* strain, reaching 1.4x10^3^ CFU/ml in non-treated mice and 1.1x10^4^ CFU/ml, an 8-fold increase, in mice treated with cyclophosphamide (**Fig 5A**). Cyclophosphamide treatment also similarly increased bacterial loads in grafts of mice infected by the WT and Δ*pilT* strains, reaching 1x10^7^ to 1x10^8^ CFU/g at 72 h PI (**Fig 5A**). In non-grafted control mice, bacteremia reached lower levels as compared to grafted mice, reflecting the need of human graft vessels for multiplication. Furthermore, no difference was observed in bacteremia between WT and Δ*pilT* infected non-grafted mice in the presence or in the absence of cyclophosphamide (**Fig 5B**). These data indicate that monocytes and neutrophils actively controlled the bacteremia of both WT or Δ*pilT* mutant in a similar manner.

Together, these data demonstrate that WT and Δ*pilT* strains triggered similar inflammatory responses and were similarly phagocytosed by phagocytic cells. This suggests that immune responses could not account for the low level of bacteremia and lethality observed in the Δ*pilT*-infected grafted mice. The data also demonstrate that sepsis-induced death is not solely the consequence of a systemic inflammatory response.

### Pilus retraction is required for bacterial release from infected microvascular endothelium

**Fig 6:**
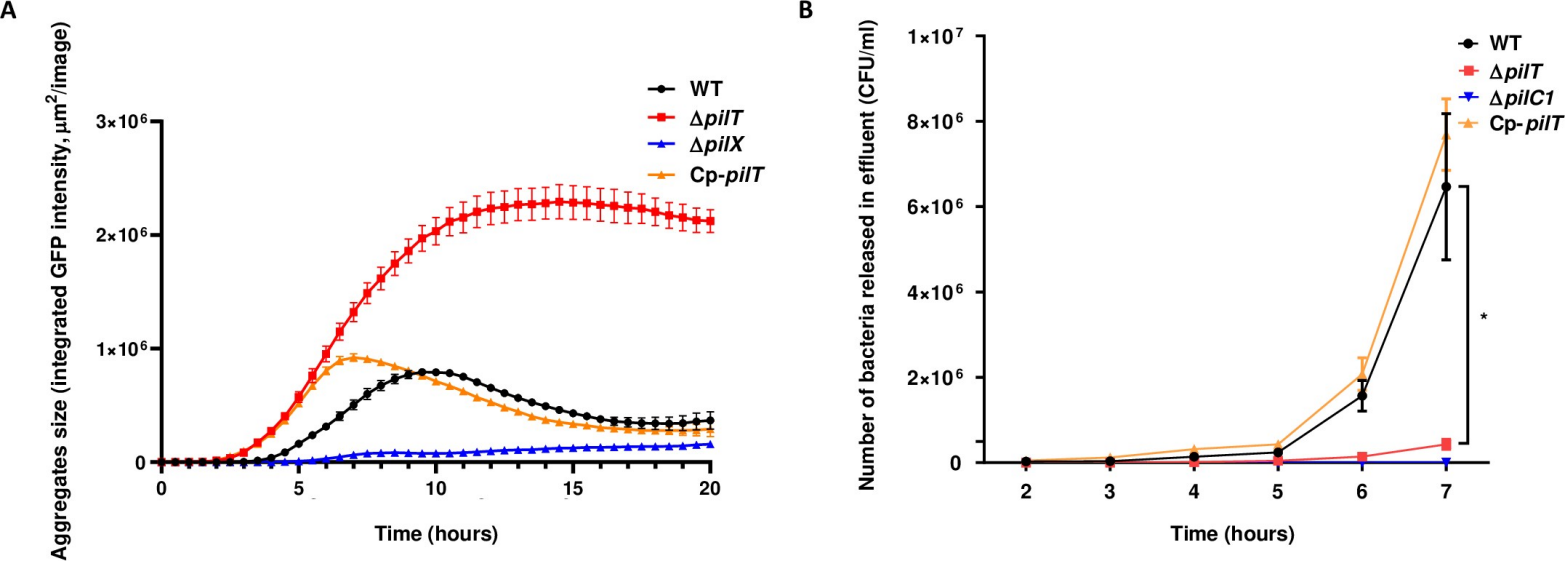
PilT-dependent pilus retraction is critical for bacterial release from microcolonies adherent to endothelial cells. **(A**) Aggregates of WT *N*. *meningitidis*, its isogenic Δ*pilT* mutant, Cp-*pilT* complemented strain and Δ*pilX* mutant producing GFP under an IPTG-inducible promoter and grown in liquid medium were imaged using the IncuCyte S3 platform at 37°C with 5% CO_2_. Four image sets from distinct regions per well were taken every 15 min using a ×20 dry objective and each condition was run in triplicate. Bacterial aggregates formation and dispersion was measured over a period of 20 h under static condition. **(B)** Number of bacteria detaching from microcolonies adhering onto human dermal microvascular cells (HDMEC) under flow condition. Cells were infected for 30 min under static condition with WT *N*. *meningitidis*, Δ*pilT* mutant, Cp-*pilT* complemented strain, and Δ*pilC1* mutant as non-adhesive control. Cells were then washed to remove non-adherent bacteria and placed under shear stress of 0.15 dyn/cm^2^. Bacterial count in the effluent was evaluated each hour by plating serial dilutions on agar plates. Three independent experiments. Bars represent mean ± SEM. * *p* < 0.05, one-way ANOVA followed by multiple comparison test.

The Δ*pilT* mutant is unable to retract TFP, is hyper-piliated and exhibits a hyper-aggregative phenotype *in vitro* [38,45]. We hypothesized that this might prevent the release of TFP-dependent contacts between bacteria and, in turn, the detachment process that releases bacteria back into the bloodstream to sustain bacteremia. To test this hypothesis, we first grew WT and Δ*pilT* strains *in vitro* in liquid medium under static condition and monitored by live microcopy the rate of bacterial aggregates formation and dispersal over a period of 20 h. As compared to a Δ*pilX* strain, an aggregation-defective mutant used as a negative control [24], both WT and Δ*pilT* strains displayed an aggregative phenotype. However, while within 10 h in culture the bacterial aggregates formed by the WT strain started to disaggregate, the Δ*pilT* aggregates were still growing and did not show signs of dispersal (**Fig 6A**). Similar to the WT strain, the complemented Cp-*pilT* strain displayed an aggregation/disaggregation phenotype, confirming that pilus retraction was required for bacteria to disaggregate.

To determine whether the lack of pilus retraction might prevent bacterial release from infected microvessels, we assessed *in vitro* the bacterial detachment from microcolonies grown at the surface of primary human dermal endothelial cells (HDMEC). During bacterial growth, numerous bacteria were released in the medium from microcolonies formed by WT meningococci whereas very few bacteria detached from Δ*pilT* microcolonies. We quantified the number of bacteria detaching from microcolonies grown at the surface of HDMEC under shear stress conditions that mimic the blood flow observed in capillaries (0.15 dyn/cm^2^) [46]. After 5 h under shear stress, WT bacteria started to significantly release from the endothelial cell surface to reach 6.5x10^6^ bacteria/ml at 7 h. Conversely, the Δ*pilT* mutant detached poorly from the infected monolayer under flow, reaching only 4.3x10^5^ bacteria/ml at 7 h, and this release was markedly delayed as compared to the WT strain (**Fig 6B**), despite having similar adhesion ability (**S5 Fig**). The complemented Cp-*pilT* strain behaved like the WT strain and as expected, no significant bacterial release was observed for the non-adhesive Δ*pilC1* control mutant, which is unable to colonize the endothelial monolayer (**Fig 6B**). These data demonstrate that Δ*pilT* bacteria poorly detach from adherent microcolonies formed on human endothelial cells and suggest that this defect also likely affects the dispersion mechanism required for a sustained bacteremia *in vivo*.

### Maintenance of a sustained bacteremia correlates with lethality in infected non-grafted mice

**Fig 7:**
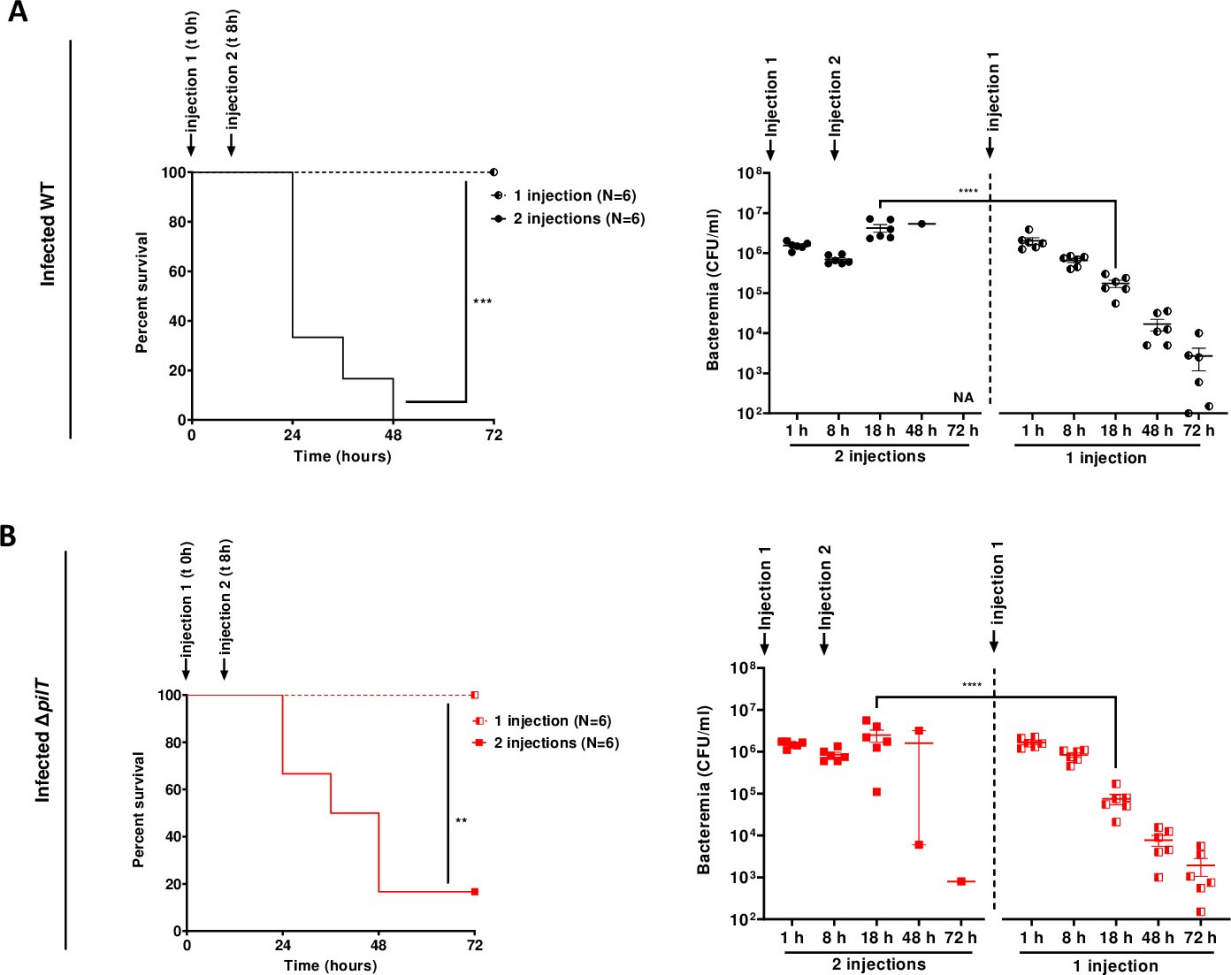
Maintenance of a sustained high bacteremia correlates with lethality. **(A, B)** Non-grafted SCID mice were infected IV with *N*. *meningitidis* WT strain (A) or Δ*pilT* mutant (B). A first infection was performed with an inoculum of 3x10^6^ CFU. A second infection with the same inoculum was performed in the 2 injections group 8 h after the first injection to mimic a prolonged bacteremia (2 injections), as control mice received one injection (1 injection). Mice survival was assessed each day during 72 h. Two independent experiments with *n* = 6 mice in each group. Kaplan-Meier plots showing the survival of infected mice (left panel, **p < 0.01; *** *p* < 0.001, two-sided log-rank Mantel-Cox analysis). Bacteremia was measured at 1, 8, 18, 48 and 72 h by culturing serial dilutions of blood samples on agar plates (right panel, bars represent mean ± SEM, **** *p* < 0.0001, one-way ANOVA followed by multiple comparison test).

Our data suggested that lethality was correlated with the ability of meningococcal strains to promote sustained bacteremia. To further investigate this correlation, we artificially maintained bacteremia above 1x10^6^ CFU/ml for both WT or Δ*pilT* mutant over a period of 18 h in non-grafted mice by injecting 3x10^6^ CFU twice with an interval of 8 h between injections (**Fig 7A and 7B**). Induced sustained bacteremia was associated with a high mortality rate between 18 h and 48 h in mice infected with both strains (lethality of 6/6 and 5/6 mice for the WT strain and the Δ*pilT* mutant, respectively) (**Fig 7A and 7B**). Hence, we establish a correlation between an artificially sustained bacteremia and lethality in mice regardless of bacterial strain used.

Taken together, our data demonstrate that pilus retraction is required to release TFP-dependent bacterial contacts and promote a continuous sustained bacteremia responsible for mice lethality.

## Discussion

TFP are major virulence factors that mediate adhesion of meningococci to human vessels and are involved in the triggering of tissue lesions. Here, we provide direct evidence that human graft microvessels confer a highly efficient replicative niche for adherent meningococci, that supports extensive bacterial multiplication. Following bacterial growth, pilus retraction is critical to release TFP-dependent bacterial contacts for bacteria to disseminate from this reservoir into the bloodstream to sustain bacteremia. As lethality was clearly associated with sustained bacteremia, we conclude that TFP retraction plays a key role in the pathophysiology and outcome of meningococcal sepsis.

TFP are widespread virulence factors among both Gram-negative and Gram-positive bacterial species, mediating physical interaction of bacteria with host cells and triggering signaling events that are essential in bacterial pathogenesis [32,47–49]. TFP biology is a complex system involving a large number of genes, many of which are conserved in both sequence and genomic organization in piliated species. In particular, PilT is highly conserved among TFP-expressing species and mediates the dynamic retraction of TFP. In many species, TFP retraction has been shown to control motility behavior, important properties of bacterial aggregates and microcolony formation [48]. Different studies have shown a role for PilT in virulence for several human or veterinary bacterial pathogens, including *Escherichia coli*, *Pseudomonas aeruginosa*, *Legionella pneumophila* or *Dichelobacter nodosus* [50–54]. In enteropathogenic *E*. *coli*, disruption of the ATP binding site of BfpF, a presumed functional homolog of PilT, reduces dispersion of bacterial aggregates and leads to a marked decrease in infectivity [50]. In *L*. *pneumophila*, an analogous change in DotB, a PilT family member based on sequence similarity, prevents the survival within macrophages [54]. Consistent with a role for pilus retraction in motility and intimate cell adhesion, a *P*. *aeruginosa* PilT-deficient strain is significantly less infectious than the WT strain in mouse models of corneal infection and acute pneumonia [51]. The *P*. *aeruginosa* Δ*pilT* mutant colonized the lungs as well as the parental strain, but exhibited a reduced ability to disseminate from the lung to the liver [51]. Few data are available for the impact of pilus-retraction on *N*. *meningitidis* virulence *in vivo*. Transgenic mice expressing human CD46, a proposed adhesion receptor for *N*. *meningitidis*, and infected with a Δ*pilT* mutant displayed a reduced bacteremia and a higher survival rate compared to the WT strain [52]. Nevertheless, the adhesion and virulence of meningococcus in this model is not TFP-dependent [55,56].

In *Neisseria* species, earlier studies performed *in vitro* have shown that inactivation of *pilT* gene results in a hyper-piliated state that is a consequence of the lack of TFP retraction. This phenotype increases binding to host cells *in vitro* [34,37,38,45]. Pilus-retraction promotes intimate adhesion leading to spreading of microcolonies onto a cell monolayer and is responsible for the full activation of the β2-adrenergic receptor that functions as a signaling receptor in infected endothelial cells [38,57,58]. In addition, it was shown that TFP retraction promotes intermittent TFP-dependent traction forces inside bacterial aggregates that control important properties of microcolonies, including viscosity, local order and shape [59,60]. *In vitro*, this property allows *N*. *meningitidis* aggregates to fill microfluidic channels, to adjust to constrained geometry and to spread onto a cell monolayer [38,40]. It has been proposed that this feature may allow meningococci to efficiently colonize the human microvasculature and to provoke capillary occlusions [40]. We show here that while the shape of the colonies formed within microvessels may vary between WT and retraction deficient stains, the number of bacteria that colonized the human vessels *in vivo* were similar, indicating that TFP retraction has no major impact on meningococcal adhesion and proliferation *in vivo*. In addition, colonization was associated with the production of equivalent cytokine levels indicating that both strains induced similar systemic and vascular inflammation. Our main observation was that loss of pilus retraction affects bacterial detachment from adherent microcolonies grown on endothelial cells, thereby reducing bacterial dissemination. These results provide evidence that TFP retraction is an essential process to release TFP-dependent contacts within bacterial microcolonies formed at the endothelial cell surface and is required for bacterial dissemination and seeding of the bloodstream. Whether the hyper-piliated state of the Δ*pilT* mutant may also participate *per se* to the lower release of bacteria observed in our *in vivo* model remains an open question. To address this point would require a strain that is as piliated as the Δ*pilT* mutant, but still able to retract pili. To our knowledge, there is no artificial means to upregulate the piliation to the level of a Δ*pilT* strain without affecting retraction.

Besides TFP, several other adhesins that are playing a role in meningococcal adhesion to host cells have been described. Among them, Opa and Opc are abundant in the outer-membrane and the best studied [61]. Unlike TFP, they are not expressed in all invasive isolates and are partially masked by the capsule, adhesion of encapsulated meningococci therefore relies on TFP [62–64]. In this study, we used Opa^-^ and Opc^-^ isolates in order to avoid interferences with these adhesins. However, *in vitro* and clinical data suggest a role for Opc in the pathogenesis of meningococcal disease. Opc has been shown to promote cell invasion and transcytosis across the brain endothelial barrier [65–67]. Interestingly, *N*. *meningitidis* strains of the hyperinvasive ET-37/ST11 clonal complex that lack the *opc* gene have been reported to cause severe cases of septicemic IMD but have a relatively low tendency to cause meningitis [68,69]. Overall, while Opc may enhance the ability to cause meningitis, this bacterial factor is not likely to play a preponderant role in vascular colonization and during the bloodstream phase of the disease.

So far, the mechanisms leading to bacterial detachment and dissemination have been poorly studied. Factors involved in bacterial adhesiveness are good candidates to influence bacterial detachment and dissemination. Among them, PilC proteins are subjected to phase variation, a mechanism enabling rapid adaptation to environmental selective pressure [70]. Because *pilC* mutants express low levels of TFP and are less adhesive, phase variation-mediated switching to OFF of PilC expression may be a means by which meningococci could detach from endothelial cells and reach the bloodstream. Genomic studies have shown that the absence of *pilC* genes expression was more frequently found in invasive rather than carriage isolates, for example in *N*. *meningitidis* serogroup W ST-11 clonal complex strains [71,72]. *In vitro*, it was shown that following adhesion to epithelial cells, a posttranscriptional modification of the major pilin PilE can occur that destabilizes TFP-dependent contacts and promotes detachment of meningococci from adherent microcolonies [73]. More recently, it was shown that lactate released by epithelial cells upon meningococcal adhesion, can act as a signal molecule that induces dispersion of bacterial microcolonies [74]. In the gonococcus, oxygen depletion promotes TFP retraction-driven microcolony disassembly *in vitro* [75]. Whether these mechanisms play a role in bacterial dissemination and disease *in vivo* remains to be determined. Here, we demonstrate that bacterial release that is promoted by TFP retraction plays a major role in maintaining sustained bacteremia. The impaired ability to maintain a high bacteremia in animals infected by the Δ*pilT* mutant was responsible for a striking decrease in virulence, as all animals survived infection. When bacteremia of the Δ*pilT* mutant was artificially maintained in non-grafted mice, it induced a similar lethality pattern as the one induced by the WT strain, indicating that a sustained high bacteremia can be solely responsible for lethal sepsis. This observation is consistent with clinical reports showing that the severity of IMD is linked to the amount of circulating bacteria in the blood [10,76].

A major question arising from this work is why and how sustained bacteremia is required to promote lethality. We subsequently addressed the role of the immune response in the control of bacteremia and lethality. The acute nature of IMD is not temporally compatible with the development of an effective adaptative immune response [10,13]. Hence, the innate immunity plays a major role in the control of meningococcal infections. This is highlighted by clinical evidences showing that immunodeficiencies increasing the risk of IMD in humans are mostly innate immune deficiencies (i.e. defects in complement, defects in inflammatory response, or spleen dysfunction) [77,78]. The human skin-grafted SCID mice therefore represent a pertinent model, since these mice have all the key effectors of the innate immune response (inflammation, complement, phagocytes, NK cells). Interestingly, we observed that infection by both WT and Δ*pilT* strains led to similar systemic and vascular inflammatory responses. This is consistent with their ability to similarly colonize the vascular wall and likely produce equivalent levels of pathogen-associated molecular patterns (PAMPs) such as the lipo-oligosaccharide (LOS), the pro-inflammatory endotoxin [79]. Hence, in our experimental conditions, a deregulated host inflammatory response was not correlated with the outcome. Until recently, the magnitude of the LOS-induced cytokine response was commonly used to assess immune function in patients undergoing critical sepsis. Plasma levels of pro-inflammatory cytokines such as TNFα, IL-1β, IL-6 and IL-8 were correlated with disease outcome [10,80–82]. However, the exact role of these cytokines in the pathogenesis of sepsis was difficult to define since their activity is modulated by the simultaneous activation of compensatory anti-inflammatory pathways shortly after sepsis initiation [83]. The hallmark cytokine is IL-10, which is produced by a variety of leukocytes, suppressing the production of IL-6 and interferon-γ (IFNγ) and stimulating the production of soluble TNF receptor and IL-1 receptor antagonists. Finally, attempts to improve survival by dampening the inflammatory response by way of IL-1 and TNF blockade had little success in reducing mortality [83], further supporting the notion that increased systemic inflammation cannot solely account for organ failure and mortality. In addition, we did not find any major differences in the cellular innate immune response to infection by the WT and Δ*pilT* strains likely to explain the stark contrast between these two strains in promoting a lethal outcome. These results indicate that, beside an overwhelming inflammation, sustained bacteremia is required to promote organ dysfunction and death. One limitation of our model is the restriction of bacterial adhesion to the human graft vasculature as the bacteria cannot interact with the microvasculature of some important organs (kidney, heart, adrenal glands) that are targets of blood-borne meningococci in patients with IMD and participate in multiple organ failures and lethality [84–86]. It is, however, likely that bacterial persistence in the bloodstream provokes profound circulatory, cellular, and/or metabolic abnormalities, associated with a greater mortality. Indeed alteration in redox balance, calcium homeostasis, endothelial integrity, energy metabolism in monocytes can all play crucial roles in sepsis progression [87–89]. Future work will aim at a better definition of the impact of sustained bacteremia in the cellular and molecular dysfunctions leading to lethal sepsis.

Altogether, this work reveals the key role of TFP retraction in the occurrence and outcome of lethal meningococcal sepsis by facilitating the release of meningococci from the vascular niche. This work furthermore highlights the role of sustained bacteremia in the pathological alterations leading to lethal sepsis.

## Material and methods

### Ethics statement

The animal experimental procedures described in this paper are in accordance with the European ethical legislation (Directive 2010/63/EU). The experimental protocol was approved by the Comité d’Expérimentation Animale de l’Université Paris Descartes (project number 12–030 and 2018012515596498). Human skin grafts were obtained from surgical waste from patients undergoing plastic surgery at Groupe Hospitalier Paris Saint-Joseph (Paris, France). According to the French legislation, the patients were informed of the research purpose and their non-opposition was orally received.

### Bacterial strains and growth conditions

*N*. *meningitidis* 8013 strain clone 2C4.3 is a piliated, adherent and encapsulated serogroup C clinical isolate that produces the class I SB pilin variant, Opa^-^, Opc^-^, PilC1^+^/PilC2^+^, as described previously [19]. The Δ*pilT* mutant was engineered by introduction of an *aph3′* kanamycin resistance cassette into the *pilT* gene locus. Chromosomal sequences upstream and downstream flanking regions of the *pilT* gene were amplified using PilT_Fr1_Fw 5’-TCAGGATGAAGTCTTGGATGG-3’ and PilT_Fr1_Rv 5’-TCAGCTCATTCACACAACCGCCTTCCGGCCATACC-3’ for upstream region and PilT_Fr2_Fw 5’-GTTCTTCTGAAATGCGGCTCTGTTTAGTATAATG-3’ and PilT_Fr2_Rv 5’-CGTCTCAATCAAAGGTTTGCCGTC-3’ primers for downstream region. PilT_Fr1_Rv and PilT_Fr2_Fw primers contained 10 nucleotides overlapping regions with the *aph3′* kanamycin-resistance cassette at their 3’ and 5’ end, respectively. The kanamycin-resistance cassette was amplified with PilT_Kn_Fw 5’-CGGTTGTGTGAATGAGCTGATTTAACAAAAATTTAAC-3’ and PilT_Kn_Rv 5’-GAGCCGCATTTCAGAAGAACTCGTCAAGAAGGCGATAG-3’ primers that contained 10 nucleotides overlapping regions with upstream and downstream flanking regions at 5’ and 3’ end, respectively. This construct was then assembled by overlapping PCR and transformed into 2C4.3 WT strain. A *pilT* complemented strain (designated Cp-*pilT)* expressing a WT *pilT* allele under the control of the strong constitutive *opaB* promotor from *N*. *gonorrhoeae* was engineered by amplifying the *pilT* allele with PilT_PacI_Fw 5’-GGCCTTAATTAAGGAGTAATTTTATGCAGATTACCGACTTACTCGCCTTCGGC-3’ and PilT_XbaI_Rv 5’-CCGGTCTAGATCAGAAACTCATACTTTCGC-3’ primers that contained overhang restriction sites for PacI and XbaI. This PCR fragment was restricted and cloned between the PacI and XbaI sites of the pMR32 plasmid encoding an erythromycin resistance marker *ermC* [90]. The complementation construct was then inserted into 2C4.3 WT strain between *trpB* and *iga* genes by transformation. The Δ*pilT* mutant kanamycin resistant construct was then introduced into the chromosome of the *pilT* complemented strain by homologous recombination. *N*. *meningitidis* Δ*pilX* and Δ*pilC1* mutants were described elsewhere [24,33]. Bacterial strains expressing the green fluorescent protein (GFP) under the control of an IPTG-inducible promoter were obtained by transformation with the pAM239 plasmid [46]. Bacterial strains were stored frozen at -80°C and routinely grown on gonococcal base (GCB) agar plates (Difco) containing Kellogg’s supplements at 37°C in moist atmosphere containing 5% CO_2_. *N*. *meningitidis* strains were selected using kanamycin at 100 mg/l and erythromycin at 3 mg/l. To select *E*. *coli* DH5α strains used for plasmid propagation, kanamycin was used at 20 mg/l.

### Mouse model of infection

Six to 8-week-old CB17/Icr-*Prkdc*^*scid*^ (Severe Combined Immunodeficiency: SCID) female mice were obtained from Janvier Labs (Saint-Berthevin, France). Mice were grafted with normal human skin as previously described [6]. Briefly, human skin was obtained from surgical wastes from patients undergoing plastic surgery. After removing adipose tissue, full thickness human skin (dermis and epidermis) was grafted onto the back of SCID mice by surgical stitching after an intraperitoneal anesthesia with ketamine 100 mg/kg, xylazine 10 mg/kg and buprenorphine 0,1 mg/kg. After a few weeks, human graft microvessels spontaneously connect with murine circulation, enabling the presence of endothelial cells of human nature in mice. Mice were infected with *N*. *meningitidis* strains grown overnight at 37°C on GCB agar plates prepared without iron (Kellogg’s supplement II) and supplemented with deferoxamine (Desferal, Novartis) at a final concentration of 15 μM. Bacterial colonies were harvested and cultured in RPMI with 1% bovine serum albumin and 0.06 μM deferoxamine under gentle agitation to reach the exponential phase of growth. Bacteria were then resuspended in physiological saline. Mice were infected intravenously and 10 mg of human holotransferrin (R&D Systems) was administered intraperitoneally just before infection. Mice that have developed signs of lethal disease were euthanized. Bacteremia was assessed by tail vein puncture. Skin-graft bacterial load was measured by crushing and homogenizing the tissue with Lysing Matrix M tubes and Fast-Prep (MP Biomedicals). Bacterial counts were determined by plating serial dilutions of the samples onto GC agar plates. The bacteria were vortexed thoroughly before plating to break up aggregates. To deplete macrophages and neutrophils, mice were treated with cyclophosphamide (C7397, Sigma-Aldrich) resuspended in physiological saline administered intraperitoneally. A first dose of 150 mg/kg was administered 4 days prior infection, a second dose of 100 mg/kg the day before infection and a third dose of 100 mg/kg 2 days post-infection when necessary.

### Bacterial detachment assay

HDMEC primary endothelial cells (Promocell) were grown in laminar flow chambers composed of six independent flow channels (μ-Slide VI 0.4, Ibidi) coated with rat tail type I collagen under 5% CO_2_ until confluence. Bacteria were grown on GCB agar plates, adjusted to OD_600_ = 0.02 in prewarmed endothelial cell medium (ECM) and cultivated for 2 hours at 37°C under shaking conditions. Cells were infected with 10^6^ bacteria (multiplicity of infection = 100) and bacteria were allowed to adhere to the cells for 30 min, then unbound bacteria were removed by three extensive washes. The number of bacteria adhering onto HDMEC at 30 min was counted after DAPI staining. Infected cells were then connected to a continuous flow of ECM applying a shear stress of 0.15 dynes/cm^2^ using a syringe pump (Harvard Apparatus). The flow chamber was placed in an incubator at 37°C with 5% CO_2_ throughout the experiment. Every hour, samples coming out of the flow chamber were collected, serial dilutions were performed and cultured on GCB agar plates to determine CFU.

### Flow cytometry

Blood was collected from deeply anesthetized mice by cardiac puncture using heparinized syringes. Spleens were removed and passed through a 70-μm filter. Red blood cells were lysed using RBC lysing buffer (Sigma). Cells were blocked using anti-CD16/32 antibody (2.4G2 clone; Bio X Cell) for 20 min on ice, then stained for 20 min with fluorescently labeled antibodies including FITC-Ly6C (HK 1.4), PE-F4/80 (BM8), PerCP Cy5.5-Ly6G (1A8), APC-Cy7-CD11b (M1/70), Brilliant Violet 421-CD45 (30-F11), and Brilliant violet 510-MHC-II (M5/114). Nonviable cells were identified using BD Horizon Fixable viability Stain. After surface staining, cells were fixed and permeabilized using Fix & Perm Cell Permeabilization kit according to manufacturer instructions (Life technologies). Phagocytosis of WT and Δ*pilT* mutant were detected by intracellular staining using a rabbit polyclonal IgG against *N*. *meningitidis* [19] and conjugated to allophycocyanin (APC) according to manufacturer instructions (Life technologies). Isotype control antibodies were used to confirm positive signals. All antibodies were purchased from Biolegend. Neutrophils were identified as CD11b^+^Ly6G^hi^. Red pulp macrophages were identified as CD11b^neg^ F4/80^hi^ and proinflammatory monocytes were identified as CD11b^+^ Ly6G^−^ Ly6C^hi^ F4/80^+^.

### Aggregates formation and dispersion assay

GFP expressing bacteria were grown in FluoBrite DMEM (Thermofisher Scientific) containing 1 mM IPTG. Bacterial aggregates were imaged using the IncuCyte S3 system (Essen Biosciences) housed within an incubator at 37°C with 5% CO_2_. Images from phase contrast and green (400-ms exposure) channels were acquired every 15 min for 18 hours using a ×20 dry objective. Four set of images were acquired per condition, and each condition was run in triplicate. Analysis and quantification were performed using IncuCyte S3 Software (Essen Biosciences).

### Multiplex cytokines immunoassays

Mice blood was centrifuged at 4000 g for 20 min at 4°C and sera were collected and frozen at −80°C. The concentrations of human and murine cytokines were quantified by electrochemiluminescence using multiplex assay kits from Meso Scale Discovery. Briefly, 25 μl of serum were added to the 96-well multi-array plates and the assays were processed following the manufacturer’s instructions. Plates were read on the multiplexing imager Sector S600 (Meso Scale Discovery).

### Statistical analysis

Statistical test is specified for each figure, *p* values of < 0.05 were considered statistically significant. Statistical analysis was performed using GraphPad Prism 8 software.

## Supporting information

S1 Fig Colonization of human skin graft by *N*. *meningitidis* WT and Δ*pilC1*. Grafted mice were infected IV with 5x10^6^ CFU of WT *N*. *meningitidis* and isogenic Δ*pilC1* mutant. Graft bacterial load at 4 hours PI was measured by quantitative culture on agar plates Two independent experiments, *n* = 6 mice per group. Bars represent mean ± SEM, ** *p* < 0.05, unpaired t-test. LOD: limit of detection.
(TIF)

S2 Fig Gating strategy used to identify splenic immune cells. Representative flow cytometry plots identifying splenic neutrophils (CD11b^+^Ly6G^hi^), red pulp macrophages, RPM (CD11b^neg^ F4/80^hi^) and inflammatory monocytes (CD11b^+^ Ly6G^−^ Ly6C^hi^ F4/80^+^) in the spleen of SCID mice.
(TIF)

S3 Fig Polyclonal rabbit antibody raised against *N*. *meningitidis* recognize efficiently phagocytized bacteria. Blood neutrophils from mice infected with WT *N*. *meningitidis* or Δ*pilT* mutant were permeabilized or not (as described in the material and methods section) and stained with an APC-conjugated rabbit polyclonal IgG against *N*. *meningitidis*. **(A)** Representative flow cytometry plots showing efficient staining in permeabilized cells. **(B)** Quantification of the staining efficiency in permeabilized and non-permeabilized neutrophils. Two independent experiments with *n* = 4 mice per group, * *p* < 0.05, two-tailed Mann-Whitney test.
(TIF)

S4 Fig Cyclophosphamide treatment depletes neutrophils, monocytes, and splenic inflammatory monocytes in SCID mice but do not affect RPM. Mice were treated with PBS or cyclophosphamide (cyclo) as described in the method section. After 24 h, blood and spleens were collected and analyzed by flow cytometry for the presence of neutrophils, monocytes and RPM. **(A)** Representative flow cytometry plots showing efficient depletion of neutrophils, monocytes, and splenic inflammatory monocytes after cyclophosphamide treatment. **(B)** Quantification of cell depletion using absolute cell counts. One experiment, *n* = 2 mice in each group.
(TIF)

S5 Fig Adhesion of *N*. *meningitidis* WT and Δ*pilT* on endothelial cells. Number of bacteria adhering onto human dermal microvascular cells (HDMEC) at 30 min under shear stress of 0.15 dyn/cm^2^. Cells were infected for 30 min with WT *N*. *meningitidis* and Δ*pilT* mutant and adhesive bacteria were counted after DAPI staining. Three independent experiments. Bars represent mean ± SEM, NS *p >* 0.05, unpaired t-test.
(TIF)
