# Supplementary figures and images for: Type IV pilus retraction enables sustained bacteremia and plays a key role in the outcome of meningococcal sepsis in a humanized mouse model
Source: PLoS Pathog. 2021 Feb 16;17(2):e1009299. doi: 10.1371/journal.ppat.1009299 (PMC7909687; doi:10.1371/journal.ppat.1009299)

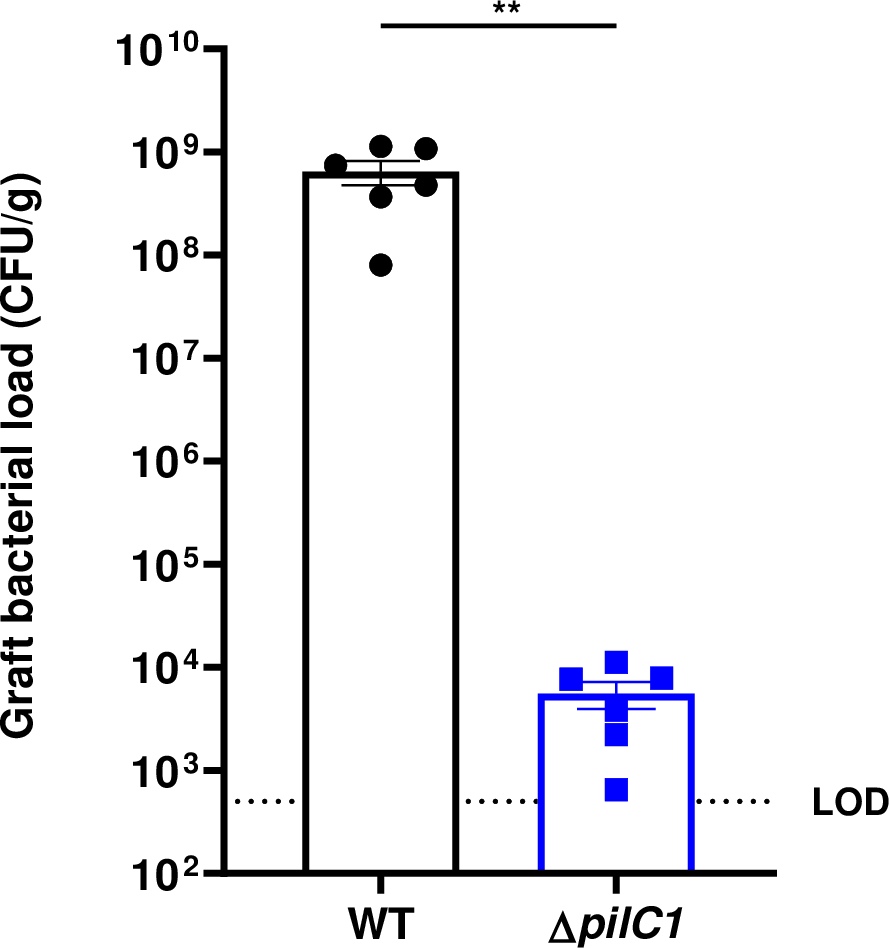

Supplement: S1 Fig — Grafted mice were infected IV with 5x10^6^ CFU of WT N. meningitidis and isogenic ΔpilC1 mutant. Graft bacterial load at 4 hours PI was measured by quantitative culture on agar plates Two independent experiments, n = 6 mice per group. Bars represent mean ± SEM, ** p < 0.05, unpaired t-test. LOD: limit of detection. (TIF) [file ppat.1009299.s001.tif]

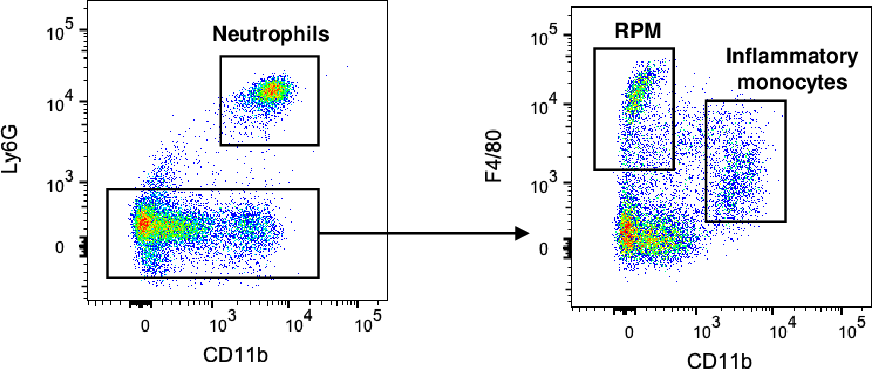

Supplement: S2 Fig — Representative flow cytometry plots identifying splenic neutrophils (CD11b+Ly6Ghi), red pulp macrophages, RPM (CD11bneg F4/80hi) and inflammatory monocytes (CD11b+ Ly6G− Ly6Chi F4/80+) in the spleen of SCID mice. (TIF) [file ppat.1009299.s002.tif]

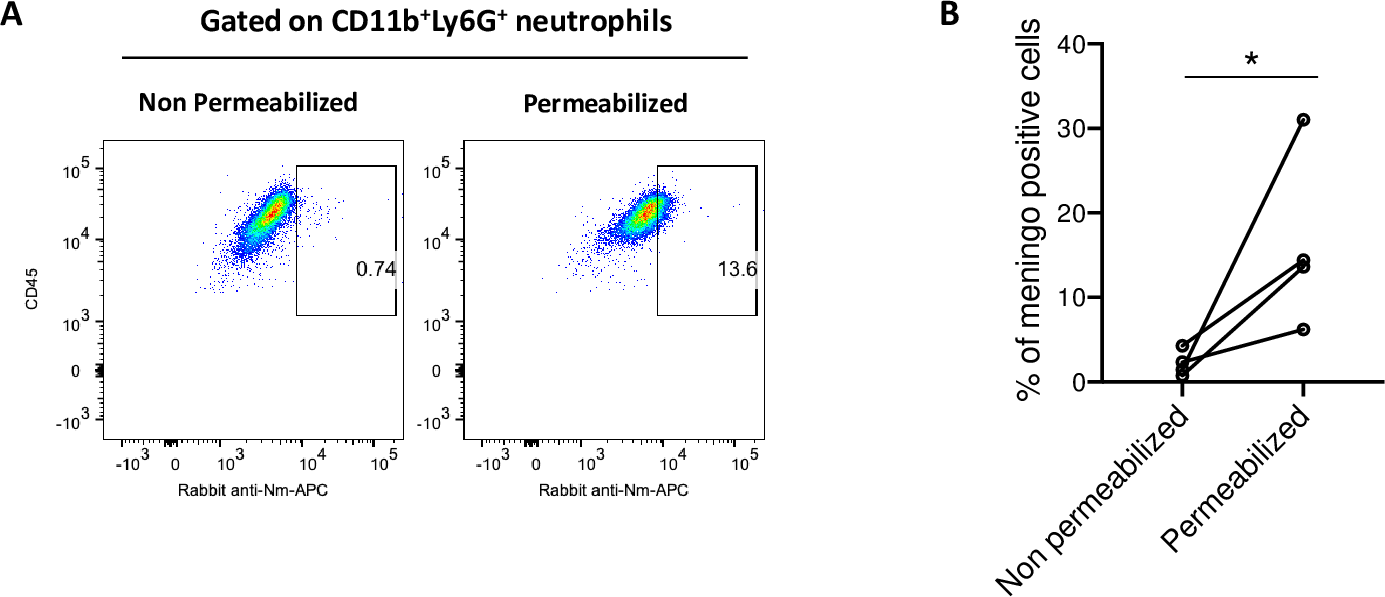

Supplement: S3 Fig — Blood neutrophils from mice infected with WT N. meningitidis or ΔpilT mutant were permeabilized or not (as described in the material and methods section) and stained with an APC-conjugated rabbit polyclonal IgG against N. meningitidis. (A) Representative flow cytometry plots showing efficient staining in permeabilized cells. (B) Quantification of the staining efficiency in permeabilized and non-permeabilized neutrophils. Two independent experiments with n = 4 mice per group, * p < 0.05, two-tailed Mann-Whitney test. (TIF) [file ppat.1009299.s003.tif]

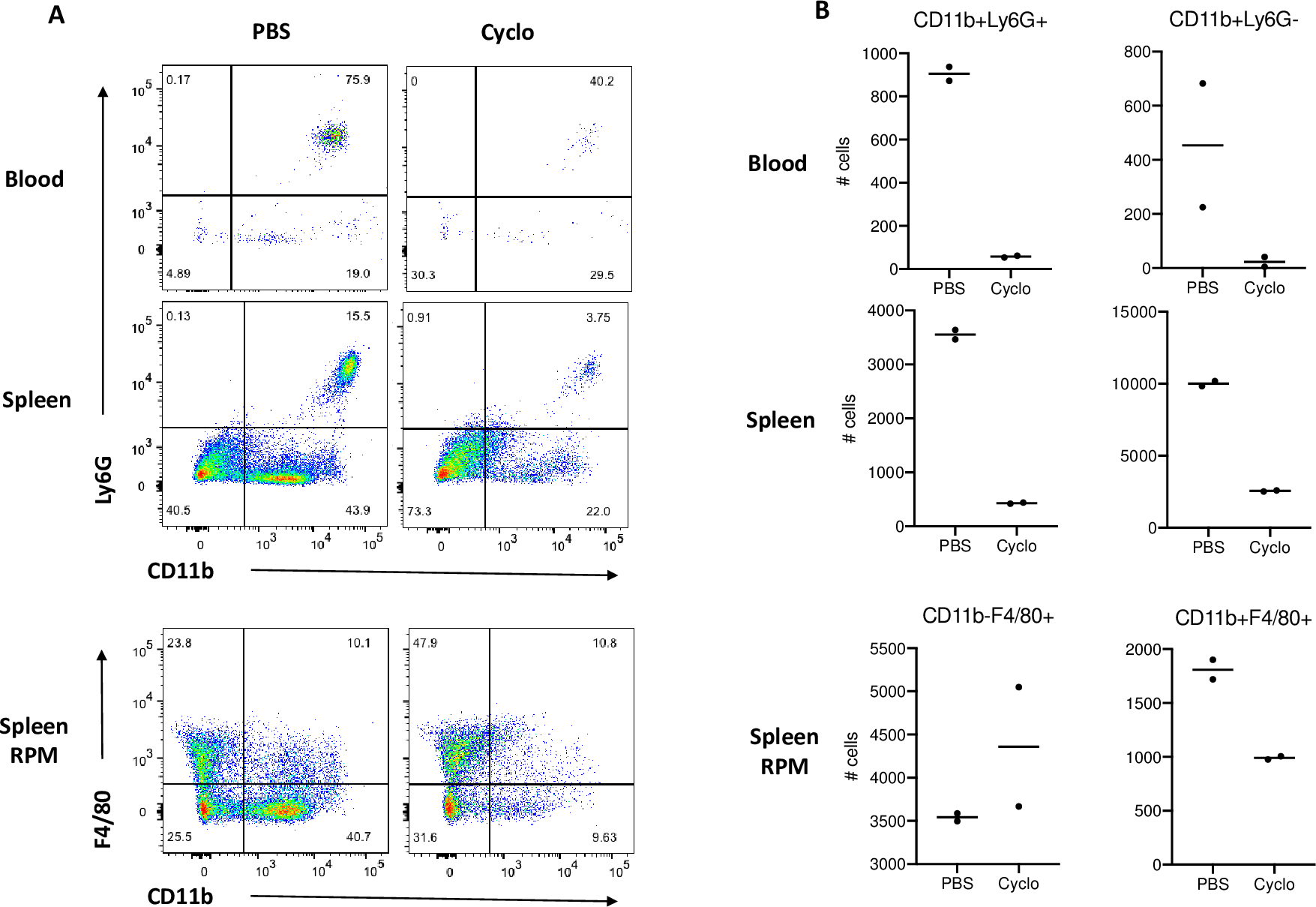

Supplement: S4 Fig — Mice were treated with PBS or cyclophosphamide (cyclo) as described in the method section. After 24 h, blood and spleens were collected and analyzed by flow cytometry for the presence of neutrophils, monocytes and RPM. (A) Representative flow cytometry plots showing efficient depletion of neutrophils, monocytes, and splenic inflammatory monocytes after cyclophosphamide treatment. (B) Quantification of cell depletion using absolute cell counts. One experiment, n = 2 mice in each group. (TIF) [file ppat.1009299.s004.tif]

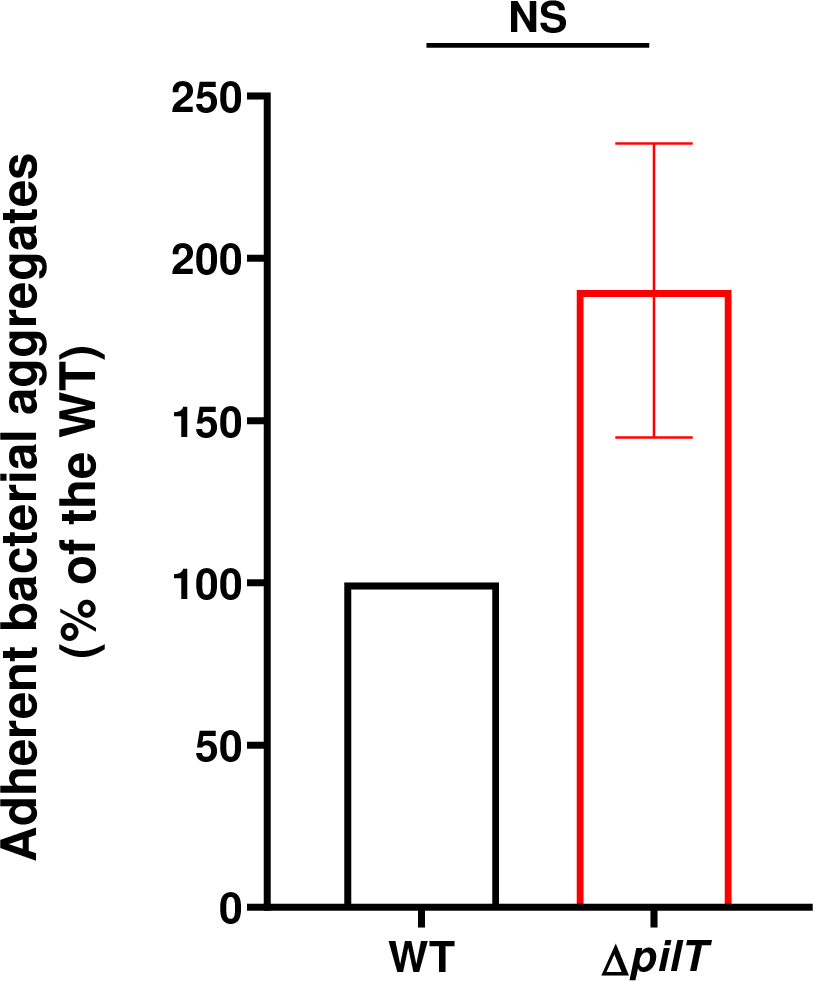

Supplement: S5 Fig — Number of bacteria adhering onto human dermal microvascular cells (HDMEC) at 30 min under shear stress of 0.15 dyn/cm2. Cells were infected for 30 min with WT N. meningitidis and ΔpilT mutant and adhesive bacteria were counted after DAPI staining. Three independent experiments. Bars represent mean ± SEM, NS p > 0.05, unpaired t-test. (TIF) [file ppat.1009299.s005.tif]
